# The Keely Treatment

**Published:** 1892-11

**Authors:** C. H. Weaver

**Affiliations:** Glenbeulah, Wis.


### The Keeley Treatment.

While a patient at the Keeley Institute, at Lancaster, you published a line from me. Now, I will further report results.

You assert that after a discontinuance of the treatment, the appetite for liquor may return. I began on ale, at the age of five years; it awoke a craving which lasted until January 15th last, when I took my last drink at the Keeley Institute—" forty-seven years of the disease."

Graduated February 4th. Am pensioned for disease of stomach and resultant nervous debility. Nervous system shattered.

Never abused myself with alcohol, but used it for its supposed tonic effects. At last, however, as is always the case, the habit grew beyond control. Came home broken down with grip and stopped the use of the remedies. I can assert, upon my sacred honor, that at no time since my return has there arisen any desire for alcoholic beverages, and that the thoughts of them causes a constantly growing feeling of repulsion.

As to the presence of atropine in the treatment, Dr. Keeley, denies it. All I know is, the eye effects are those produced by atropine, but I can assert this: I have not seen a patient hurt by the treatment, and even if it did crowd a broken down man a little hard occasionally, think what the treatment does—frees a man from the "demon desire," and a man who wants to get free should be willing to take more chances than the Keeley treatment calls for.

Welcome, even atropia, if it will knock out whisky. If you want to do a good act for humanity, judge the Keeley treatment by its results. I know nothing of its components, don't want to, but do know that I am cured, body and mind, of the faintest desire for the old enemy. C. H. WEAVER, M.D.

Glenbeulah, Wis.

